# Semi-automated brain tumor segmentation on multi-parametric MRI using regularized non-negative matrix factorization

**DOI:** 10.1186/s12880-017-0198-4

**Published:** 2017-05-04

**Authors:** Nicolas Sauwen, Marjan Acou, Diana M. Sima, Jelle Veraart, Frederik Maes, Uwe Himmelreich, Eric Achten, Sabine Van Huffel

**Affiliations:** 1 Department of Electrical Engineering (ESAT), STADIUS Centre for Dynamical Systems, Signal Processing and Data Analytics, KULeuven, Kasteelpark Arenberg, Leuven, Belgium; 2 imec, Kapeldreef 75, Leuven, 3001 Belgium; 3 Department of Radiology, Ghent University Hospital, De Pintelaan 185, Ghent, 9000 Belgium; 4 Department of Physics, iMinds Vision Lab, University of Antwerp, Edegemsesteenweg 200–240, Antwerp, 2610 Belgium; 5 Department of Electrical Engineering (ESAT), PSI Centre for Processing Speech and Images, KULeuven, Kasteelpark Arenberg 10, Leuven, 3001 Belgium; 6 Department of Imaging and Pathology, Biomedical MRI/MoSAIC, KULeuven, Herestraat 49, Leuven, 3000 Belgium

**Keywords:** MRI, Segmentation, Brain tumors, Non-negative matrix factorization, Unsupervised classification

## Abstract

**Background:**

Segmentation of gliomas in multi-parametric (MP-)MR images is challenging due to their heterogeneous nature in terms of size, appearance and location. Manual tumor segmentation is a time-consuming task and clinical practice would benefit from (semi-) automated segmentation of the different tumor compartments.

**Methods:**

We present a semi-automated framework for brain tumor segmentation based on non-negative matrix factorization (NMF) that does not require prior training of the method. L1-regularization is incorporated into the NMF objective function to promote spatial consistency and sparseness of the tissue abundance maps. The pathological sources are initialized through user-defined voxel selection. Knowledge about the spatial location of the selected voxels is combined with tissue adjacency constraints in a post-processing step to enhance segmentation quality. The method is applied to an MP-MRI dataset of 21 high-grade glioma patients, including conventional, perfusion-weighted and diffusion-weighted MRI. To assess the effect of using MP-MRI data and the L1-regularization term, analyses are also run using only conventional MRI and without L1-regularization. Robustness against user input variability is verified by considering the statistical distribution of the segmentation results when repeatedly analyzing each patient’s dataset with a different set of random seeding points.

**Results:**

Using L1-regularized semi-automated NMF segmentation, mean Dice-scores of 65%, 74 and 80% are found for active tumor, the tumor core and the whole tumor region. Mean Hausdorff distances of 6.1 mm, 7.4 mm and 8.2 mm are found for active tumor, the tumor core and the whole tumor region. Lower Dice-scores and higher Hausdorff distances are found without L1-regularization and when only considering conventional MRI data.

**Conclusions:**

Based on the mean Dice-scores and Hausdorff distances, segmentation results are competitive with state-of-the-art in literature. Robust results were found for most patients, although careful voxel selection is mandatory to avoid sub-optimal segmentation.

## Background

High-grade gliomas (HGGs) account for 80% of all malignant primary brain tumors [1]. Standard treatment of HGG consists of complete or partial resection of the gross tumor volume, followed by radiotherapy and/or chemotherapy to attack the remaining tumor cells and to counteract tumor recurrence. So far, recent advancements in glioma research have only had little effect on patient outcome. The 5 year survival rate of the most common histopathological subtypes, anaplastic astrocytoma and glioblastoma multiforme (GBM) are 26 and 5%, respectively [1].

MRI has become the imaging modality of choice for evaluating tumor progression and the efficacy of a chosen treatment strategy. GBM, the most malignant and most common type of glioma, tends to invade the healthy tissue, such that tumor margins extend beyond the imageable component of the tumor based on conventional MRI (cMRI). Numerous recent studies have recommended incorporating additional imaging biomarkers from advanced MRI modalities such as perfusion-weighted imaging (PWI) and diffusion-weighted imaging (DWI) [2–4]. PWI is used for studying tumor angiogenesis, as HGGs are known to stimulate vascular ingrowth [5]. PWI measurements in the peritumoral region have been reported to differentiate GBM from metastases, suggesting the detection of tumor infiltration [6]. PWI was also found useful in differentiating tumor recurrence from radiation necrosis [7]. DWI assesses the mobility of water molecules within tissue due to Brownian motion. Because glioma infiltration disrupts the organization of the white matter tracts, DWI is potentially useful to characterize the extent of infiltration. Diffusion tensor imaging has been reported to better delineate tumor margins in gliomas than cMRI [8].

Assessment of tumor extent plays a key role at all stages of the treatment process. An outline of the gross tumor volume is made upon planning surgical resection and for defining the radiotherapy target volume. During follow-up, the response assessment in neuro-oncology (RANO) criteria are applied for monitoring tumor growth, assessing the maximal diameter of the lesion in 2 orthogonal directions on axial slices [9]. Clinical practice would benefit from volumetric measurements of the tumor and its subcompartments. With the recent emergence of the dose-painting concept in radiotherapy, segmentation of the active tumor region would allow focusing the radiation energy to the most active part of the tumor in a non-uniform way [10]. Volume measures of the active tumor and necrotic region have been reported to be significant predictors of patient outcome to treatment [11, 12]. Furthermore, the RANO criteria acknowledge that volumetric measurements might be favorable compared to cross product diameters in cases of irregularly shaped tumors, multi-focal tumors and tumors with cystic or necrotic components. However, segmentation of HGGs in MRI is a challenging task, due to their heterogeneous nature: several stages of the disease can occur throughout the same lesion and diffuse boundaries exist between active tumor, necrosis, edema and the surrounding healthy brain. Manual segmentation of 3D images is also time-consuming, which is the main reason why volumetric tumor delineation has not become widespread in clinical practice.

In recent years, significant advancements have been made in the field of automated brain tumor segmentation. Both semi-automatic and fully automatic methods have been proposed [13]. A popular approach is to combine imaging biomarkers from different MRI sequences on a voxel-wise basis, thereby increasing specificity and reducing overlap between tissue classes. Nowadays, supervised classification methods are receiving most attention [14]. These methods rely on an extensive set of training images with manually annotated ground truth to learn decision boundaries between the tissue classes in feature space. The most popular methods include random forests [15, 16], support vector machines [17, 18] and neural networks [19]. Additional constraints may be imposed to further enhance performance, such as spatial consistency of the tissue regions [20, 21].

Unsupervised classification methods are also being considered for tumor segmentation. These methods are very flexible, as they don’t require an extensive training dataset with a uniform acquisition protocol. They learn classification rules directly from the imaging data at hand, based on some similarity criterion. Popular approaches include fuzzy C-means clustering (FCM) [22, 23], Gaussian mixture modeling [24, 25], hidden Markov Random Fields [26, 27] and non-negative matrix factorization (NMF) [28, 29]. Due to the absence of training data, unsupervised methods rely more strongly on the incorporation of prior knowledge to obtain competitive results. As gliomas exhibit a wide variability in terms of size, shape, location and appearance, imposing proper prior knowledge is difficult. Several studies make use of a normal brain atlas to differentiate the pathological region from the healthy tissue structures [25, 30]. This approach assumes that the healthy brain structures are not altered by the tumor, which might not be valid for large tumors deforming the surrounding healthy tissue. Some studies assume the active tumor region to be either enhancing [30] or non-enhancing [22], or hyper-perfused [23]. These assumptions do not hold in general in heterogeneous gliomas with varying degrees of contrast enhancement and/or perfusion. Another approach is to detect tumors by looking at the dissimilarity across the hemispheres, thereby supposing that the tumor volume is restricted to one of the hemispheres [31]. For the removal of false positives, several segmentation algorithms only retain the largest connected component in the segmentation mask, thereby assuming only one tumor volume [22, 32]. Evidently, many unsupervised segmentation studies incorporate prior knowledge in the form of simplifying rules, limiting their applicability to a subset of gliomas with specific characteristics. Some methods are limited to a particular set of MRI parameters, as they rely on one or more specific imaging biomarkers.

Semi-automated segmentation algorithms incorporate prior knowledge in the form of user-specific input, either as an initialization or as a post-processing step. Competitive performance has been reported for semi-automated algorithms [14]. Kwon et al. combined a normal brain atlas with a tumor growth model [33]. User-defined seeding points are used to initiate the tumor growth model, allowing to model multi-focal gliomas as well. Hamamci et al. initialized a cellular automata algorithm based on the maximal tumor diameter as drawn by the user on T1C images [34]. An active level-set surface is then initialized from the user-defined maximal diameter, to impose spatial smoothness on the contour of the pathological region. Havaei et al. initialized a k-nearest neighbors (kNN) classifier with user-selected voxels in the tumor subcompartments as well as in the healthy brain regions [35]. Spatial coordinates of the seeding points were also exploited in the feature set. The main drawback of semi-automated methods is that they don’t provide reproducible results, as the segmentation depends on subjective user input. Robustness against user input variability is therefore a key aspect of these methods. In the current study, we propose a semi-automated brain tumor segmentation method based on regularized non-negative matrix factorization. User-defined seeding points in the pathological regions are combined with a sophisticated seeding method for the normal brain tissues to initialize the NMF algorithm. Piece-wise spatial smoothness as well as sparseness of the NMF tissue abundance maps are encouraged through L1-regularization. Morphological post-processing based on the spatial location of the user-defined seeds is exploited to further remove false positives. The proposed method is applicable to all types of gliomas with any MP-MRI dataset, as the user-defined prior knowledge is patient-specific. We illustrate segmentation performance on an MP-MRI dataset of 21 HGGs combining cMRI, PWI and DWI. To verify robustness against user input variability, each patient’s dataset is repeatedly analyzed with randomly selected seeding points from the pathological subcompartments.

## Methods

### Patient population

Twenty-one patients who were diagnosed with a HGG were enrolled in the study: 1 grade II astrocytoma with focal progression to grade III anaplasia, 2 grade II oligodendrogliomas with focal progression to grade III, 1 oligo-astrocytoma with focal progression to grade III, 6 anaplastic oligo-astrocytomas, 1 anaplastic astrocytoma with focal progression to GBM and 10 GBMs. The Ghent University Hospital local ethics committee allowed a retrospective analysis of the data.

### Multi-parametric MRI dataset

The MR examinations were performed on a 3T Siemens Trio Tim scanner (Erlangen, Germany), using a standard 12-channel phased array head coil. All patients underwent an MP-MRI acquisition protocol, consisting of cMRI, PWI and DWI.

cMRI consisted of a 3-dimensional T1-weighted gradient-echo sequence (MPRAGE) before and after contrast administration, with isotropic voxels, and a 3-dimensional T2-weighted inversion recovery sequence (FLAIR) with isotropic voxels. An overview of the image acquisition settings which are defined for all MRI modalities is given in Table 1.

**Table 1:** Overview of the MR acquisition parameters for cMRI, PWI and DWI

		TR [ms]	TE [ms]	TI [ms]	voxel size [mm ^3^]	Field of view [mm ^2^]	Flip angle [°]
cMRI	T1/T1C	1550	2.39	900	0.9 ×0.9×0.9	220×220	9
	FLAIR	6000	421	2100	1.0 ×1.0×1.0	250×234.5	120
PWI		1000	29	–	1.8 ×1.8×1.8	230×230	90
DWI		5400	80	–	2.0 ×2.0×3.0	264×264	90

PWI was performed by using a lipid-suppressed, T2*-weighted echo-planar imaging sequence. A series of 90 multi-section acquisitions was acquired at 1 second intervals. The first 10 acquisitions were performed before contrast agent injection to establish a pre-contrast baseline. At the tenth acquisition, a 0.1 mmol/kg body weight bolus of gadobutrol (Gadovist, Bayer) was injected with a power injector (Spectris, Medrad Inc., Indianola, PA) at a rate of 4 ml/s through a 18-gauge intravenous catheter, immediately followed by a 20 ml bolus of sodium chloride solution at 4 ml/s. Relative cerebral blood volume (rCBV) maps were derived from the dynamic signal intensity curves in the DSCoMAN software (Dynamic Susceptibility Contrast MR Analysis, Duke University, Durham, NC). DSCoMAN computes rCBV based on the method proposed by Boxerman et al. [36], compensating for contrast agent leakage due to disruption of the blood-brain barrier. The measured relaxivity change in each voxel is approximated as a linear combination of the whole-brain average relaxivity change in non-enhancing voxels, $\overline {\Delta R_{2}^{*}(t)}$, and its time integral:

1 $$ \Delta R_{2}^{*}(t)\approx{K_{1}\overline{\Delta R_{2}^{*}(t)}-K_{2}\int_{0}^{t} \overline{\Delta R_{2}^{*}(\tau)}d\tau}   $$

where $\Delta R_{2}^{*}(t)$ represents the measured relaxivity change in a voxel. The first term reflects the uncontaminated relaxivity change and the second term reflects the effects of leakage. Linear least squares fitting is applied to compute the weighting factors *K*_1_ and *K*_2_ over all the voxels. Corrected relaxivity curves are obtained by only withholding the *K*_1_-term.

Axial diffusion-weighted images were acquired using a fast single-shot gradient-echo echo-planar imaging sequence with diffusion gradient *b*-values of 0, 500 and 1000 s/mm ^2^. The b500 and b1000 images were acquired in 3 orthogonal directions. An affine coregistration was applied to account for eddy currents. Apparent Diffusion Coefficient (ADC) maps were derived from the 3 *b*-values using weighted linear least squares fitting [37]:

2 $$ \beta = (X^{T}WX)^{-1}X^{T}Wy   $$

where *β* is the diffusion model’s parameter vector, containing the ADC value, *X* is the design matrix of all diffusion gradients, and *y* is a vector containing the logarithm of the signal intensities. *W* is a weighting matrix to take into account the heteroscedasticity, i.e. the fact that the lower signals in *y* have a higher variance, as a result of the logarithmic transform:

3 $$ W = diag(exp(2X\beta_{LLS}))   $$

where *β*_*LLS*_ represents the initial estimate of *β* obtained using standard linear least squares fitting. The raw b0 images were also added to the input dataset, serving as a T2-weighted reference.

Six MRI features were obtained from the raw acquired data after pre-processing: T1, T1C, FLAIR, rCBV, ADC and b0. All MP-MRI features were coregistered and resampled to the same spatial resolution of 1×1×3 *mm* ^3^. cMRI data were skull-stripped and T1C served as a reference for rigid coregistration in SPM8 (Wellcome Trust Centre for Neuroimaging, University College London), using the normalized mutual information criterion [38] and cubic B-spline interpolation for reslicing. Analyses were performed on 10 axial slices located around the tumor centre. Additional intensity-based features were added to the feature set to include localized spatial information. An in-plane local neighborhood of 3×3 and 5×5 voxels was used to calculate average intensity values that were assigned to the central voxel. These spatially averaged intensity values were added for all MP-MRI features. The averaged features were added in accordance with a previous study, in which it was shown that their inclusion significantly improved MRI-based brain tumor segmentation [28]. The same finding was also confirmed for the current study. Each feature’s full range was rescaled linearly to [0–1]. A total of 18 MRI features was finally obtained, making up the rows of the input matrix *X*.

### Segmentation framework

#### Non-negative matrix factorization

NMF provides a rank-*r* approximation of a non-negative input matrix *X* by the product of 2 non-negative factor matrices, *W* and *H*:

4 $$  X\approx{WH} \;\;\text{with}\quad X\in{R^{m\times{n}}_{+}}, W\in{R^{m\times{r}}_{+}} \quad\text{and}\quad H\in{R^{r\times{n}}_{+}}  $$

with *m* being the number of input features and *n* the number of data points in *X*. NMF reveals an additive parts-based structure of the input data. It represents each column of *X* by a weighted sum of the *r* columns of *W*. As we are dealing with image intensities, the non-negativity constraint applies naturally. Each column of *X* corresponds to one voxel’s MP-MRI feature set. Each column of *W* represents a tissue-specific signature, i.e. an MP-MRI feature vector corresponding to one pure tissue type. As such, each voxel’s MP-MRI feature vector is approximated as a weighted sum of (tissue-specific) source vectors. Each column of *H* represents the weights of the tissue types for one voxel. One row of *H* contains the abundances of one particular tissue type over all the voxels, which can be transformed back into the image space to obtain a tissue abundance map. The following objective function is considered for solving the NMF problem:

5 $$   \min_{W,H}f(W,H)=\min_{W,H}\quad\frac{1}{2}\left(\Vert{X-WH}\Vert^{2}_{F}+\lambda\Vert{(L+I)H}\Vert_{1}\right)  $$

The objective function consists of 2 terms. The first term minimizes the difference between the input matrix *X* and its factorization, *WH*, based on the Frobenius norm. The second term is an L1-regularization term, which consists of 2 components. The first component, *LH*, promotes piece-wise smoothness on the tissue abundance maps. *L* is a sparse *n* ×*n* matrix, with each row containing a vectorized Laplacian kernel. As such, each row of *L* applies a two-dimensional second order spatial derivative to the corresponding voxel. An in-plane neighborhood of 4 voxels is considered for the Laplacian kernel. Due to the relatively low out-of-plane resolution of the MRI data, neighboring voxels in adjacent slices are not considered. The second component applies L1-regularization to *H* directly, imposing sparseness to the abundance maps. The above NMF formulation [39] is solved using the structured data fusion framework [40] as implemented in Tensorlab [41]. A transformation of variables is used to convert the constrained optimization problem in Eq. 5 into an unconstrained problem, by squaring the entries of the factor matrices. The Gauss-Newton algorithm with dogleg trust region (GNDL) is used to solve the resulting non-linear least-squares problem [42]. At each iteration of the GNDL algorithm, a step is calculated by iteratively solving a linearized version of Eq. 5 using conjugate gradients. A maximum number of 500 iterations and a convergence tolerance of 10^−6^ for the relative difference of two subsequent values of the objective function were used as stopping criteria for the NMF analyses. The regularization coefficient *λ* was empirically set to 0.1, after testing a range of *λ* values and refining the search near *λ*=0.1.

#### NMF initialization

As NMF poses a non-convex optimization problem, initialization of the factor matrices (*W*_0_ and *H*_0_) is required. Careful initialization is important, as it might speed up the convergence process and influence the final segmentation result. The pathological tissue sources are initialized based on voxel selection. The user is required to select one or more voxels in each pathological region, i.e. active tumor and, if present, necrosis and edema. For each selected voxel, a candidate source vector is added to *W*_0_ as the averaged feature vector of the selected voxel and its 4 in-plane neighboring voxels. For each pathological tissue class, we then calculate the correlation coefficient between the candidate source vectors as their inner vector product after normalization. The initial pathological sources are obtained after merging candidate source vectors with a correlation coefficient higher than 0.95 in *W*_0_ and replacing them by their average, thereby reducing complexity of the NMF model.

As the normal brain tissue types (i.e. white matter, grey matter, cerebro-spinal fluid and blood vessels) are still highly abundant in the affected brain, an automated seeding procedure is used to obtain their initial source vectors. To cope with the variance within the tissue classes, 2 sources are assigned to each normal tissue type, leading to a total of 8 normal tissue sources. We use the Successive projection algorithm (SPA) [43] to obtain an initial estimate of the normal sources. SPA returns a subset of voxels from the input matrix *X* with minimal collinearity in feature space. All columns of *X* are first projected onto the orthogonal subspace of the already initialized pathological sources. A first normal seeding point is selected as the voxel with the highest L2-norm in the orthogonal subspace. All columns are then further projected onto the orthogonal subspace of the added voxel, and the next seeding point is selected as the point with the highest L2-norm among these projected columns. The above procedure is repeated until 8 voxels (for initializing the 8 normal tissue sources) have been selected. Similarly to the pathological sources, the normal sources are obtained as the averaged feature vector of each selected voxel and its 4 in-plane neighboring voxels, assuming these to belong to the same tissue type as the selected voxel. As SPA is known to be sensitive to outliers [44], an additional FCM procedure is applied, using the initial pathological sources and the normal sources obtained from SPA to initialize the cluster centroids. FCM alternatingly updates the cluster centroids and the cluster membership values. As we already obtained proper initialization of the pathological tissue types through user input, the pathological cluster centroids are forced to remain the same throughout the FCM updating procedure. The final FCM centroids make up the columns of *W*_0_. The columns of *H*_0_ are found by applying non-negative least squares fitting to the corresponding column of X and *W*_0_. To reduce computation time, a downsampling factor of 2 was applied to the x- and y-direction of each image slice. After NMF analysis, the obtained tissue abundance maps were upsampled again through linear interpolation. Performance was not affected by this downsampling procedure.

#### Morphological post-processing

After NMF analysis, each voxel is assigned to the source for which it has the highest abundance value. Voxels that were assigned to different sources belonging to the same pathological tissue class are merged to obtain a preliminary tissue segmentation mask. A morphological post-processing procedure, consisting of 2 steps is then applied to the pathological segmentation masks to further remove false positives, by exploiting the known location of the user-selected voxels. Spatial consistency of the tissue regions is assumed, therefore the segmentation masks are analyzed in terms of (3D-)connected components. For each selected pathological tissue type, only the connected component closest to each user-defined voxel is withheld in the corresponding tissue segmentation mask (Step 1). As the user-defined voxels are not necessarily selected close to the centre of the tissue region, the distance between a selected voxel and a connected component is defined as the minimal distance between the selected voxel and any voxel from the connected component. It is possible that the same connected component is found for several or all user-selected voxels of the same tissue class.

**Fig. 1:**
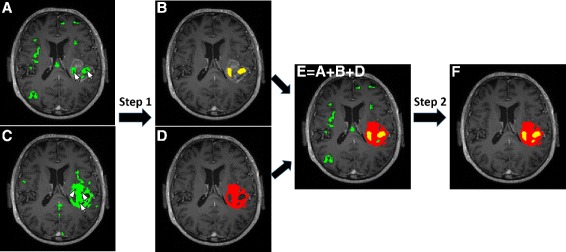
Illustration of morphological post-processing after initial semi-automated NMF based segmentation of necrosis **a** and active tumor **c**. Step 1: false positives are removed by withholding only the connected components closest to the user-defined seeding points (marked by cursor arrows) for necrosis (**b**) and for active tumor (**d**). Step 2: spatial adjacency of the connected components in the preliminary necrosis mask (*green*) to the withheld active tumor mask (*red*) is verified in (**e**). The final necrosis mask is shown in yellow in (**f**)

To avoid missing disjoint regions of a tumor component for which no voxels were selected by the user, an additional step is performed which assumes spatial connectivity of the various pathological components (Step 2). For instance, necrotic areas are always adjacent to an active tumor region. Therefore, any connected component of the preliminary necrosis mask which has a common edge with any withheld active tumor component is also included. Similarly, components of the preliminary active tumor mask are also included if they have a common edge with any withheld necrotic component. The same procedure is also applied to edema, by verifying spatial adjacency to the active tumor region(s). Figure 1 illustrates the morphological post-processing procedure for the necrotic tissue mask. One small necrotic component is not withheld after Step 1, as the user didn’t click in this region. However, it was still recovered in Step 2 by verifying spatial connectivity with the active tumor mask.

### Validation

Segmentation results of the pathological tissue regions were compared against manual segmentation by an experienced radiologist. The Dice-score was used to quantify the spatial alignment between semi-automated and manual segmentation:

6 $$ Dice_{\text{{tissue}}}=2\times{\frac{A_{\text{{tissue,NMF}}}\cap{A_{\text{{tissue,man}}}}}{A_{\text{{tissue,NMF}}}+{A_{\text{{tissue,man}}}}}}  $$

where *A*_tissue,NMF_ is the area segmented by NMF and *A*_tissue,man_ the area manually segmented by the radiologist for the same tissue type. Additionally, the Hausdorff distance was calculated for evaluating the distance between segmentation boundaries. The Hausdorff distance is the maximum distance of all points from one segmentation mask to the corresponding nearest point of the other segmentation mask:

7 $$  \begin{aligned} Haus_{\text{{tissue}}}&= max\left(\sup_{p\in A_{\text{{tissue,NMF}}}} \inf_{t\in A_{\text{{tissue,man}}}}d(p,t), \sup_{t\in A_{\text{{tissue,man}}}}\right.\\ &\qquad\qquad\left.\inf_{p\in A_{\text{{tissue,NMF}}}}d(t,p)\right) \end{aligned}  $$

where *sup* and *inf* represent the supremum and infinum, respectively. *d*(*p*,*t*) is a distance metric, for which the Euclidean distance is commonly used. The Hausdorff distance is however susceptible to small outlying subregions in either segmentation masks, as it considers the maximum surface distance. To overcome this limitation, we considered a more robust version of the Hausdorff measure, reporting the 95-percentile instead of the maximum surface distance. In analogy to previous work [14], Dice-scores and Hausdorff distances are reported for active tumor, the tumor core (active tumor + necrosis) and the whole tumor (tumor core + edema). To verify robustness of the semi-automated method to user input variability, the NMF analysis was repeated 20 times per patient with different selection of the seeding points. In each run, an automatic random selection of 3 points was performed in each pathological region, based on the manual segmentation masks.

Besides NMF with spatial regularization and sparseness ($\mathbf {NMF_{\text {spatial\_sparse}}}$), additional NMF analyses were performed to assess the added value of the regularization term and the advanced MRI modalities: NMF without regularization ($\mathbf {NMF_{\text {no\_reg}}}$), NMF with only spatial regularization (**N** **M** *F*_spatial_), NMF with spatial regularization and sparseness but without the morphological post-processing step ($\mathbf {NMF_{\text {no\_postproc}}}$), and NMF with spatial regularization and sparseness when only considering cMRI data (**N** **M** *F*_cMRI_). For the **N** **M** *F*_cMRI_ analyses, rCBV and ADC were omitted from the MP-MRI feature set but b0 was withheld, acting as a surrogate for *T*_2_-weighted MRI.

## Results

**Fig. 2:**
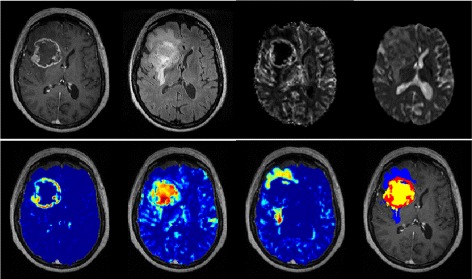
*First row*: coregistered MP-MRI maps of a GBM patient, *left to right*: T1C, FLAIR, rCBV, ADC. *Second row, left to right*: NMF abundance maps for active tumor, necrosis and edema. The final segmentation masks are shown on the right for active tumor (*red*), necrosis (*yellow*) and edema (*blue*)

Figure 2 gives an example of the regularized NMF results and tissue segmentation for a GBM patient with a typical ring-enhancing lesion. Some of the MP-MRI input maps are shown on the first row. The NMF abundance maps as well as the final segmentation masks are shown for the pathological tissue types on the second row.

**Fig. 3:**
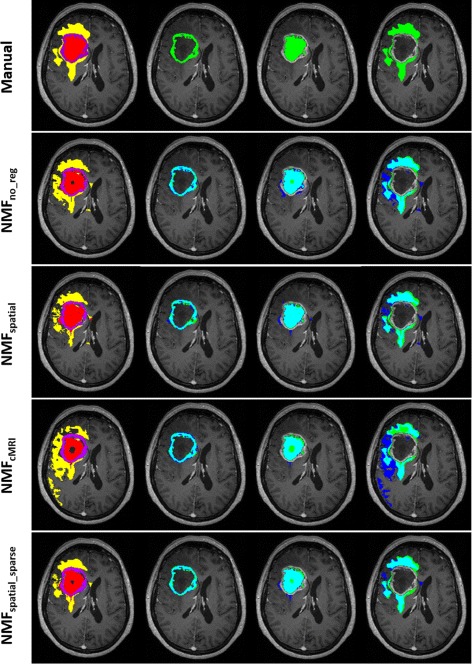
Comparison of the segmentation results of the pathological tissue regions obtained for a GBM patient using the different NMF methodologies. The *left figure* of each row shows the obtained segmentation for active tumor (*purple*), necrosis (*red*) and edema (*yellow*). The *second to fourth figure* of each row show the individual segmentation for active tumor, necrosis and edema, respectively. The *top row* shows manual segmentation, whereas the other rows show the overlap between the NMF (*blue*) and manual (*green*) segmentation result. Segmentation overlap is marked in *cyan*

Figure 3 gives a comparison of the segmented pathological regions for a GBM patient obtained from the different NMF analyses ($\mathbf {NMF_{\text {no\_reg}}}$, **N** **M** *F*_spatial_, **N** **M** *F*_cMRI_ and $\mathbf {NMF_{\text {spatial\_sparse}}}$) and manual segmentation. For the active tumor region, spatial overlap with manual segmentation is slightly lower for **N** **M** *F*_spatial_ compared to the other methods. On the other hand, the spatial regularization term did allow **N** **M** *F*_spatial_ to segment the entire necrotic region, whereas the other methods missed a central portion of the necrotic area. Segmentation of edema is inferior for **N** **M** *F*_cMRI_, where the differentiation from surrounding healthy brain structures was found difficult. $\mathbf {NMF_{\text {spatial\_sparse}}}$ shows the least false positive regions for necrosis and edema compared to the other methods.

**Fig. 4:**
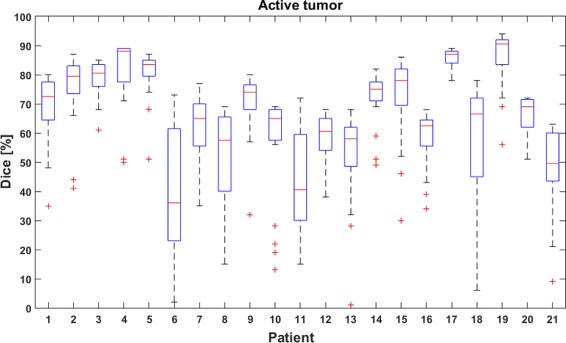
Boxplots showing the dispersion of the Dice-scores for active tumor. Boxplots show quartile ranges of the Dice-scores, ’+’ indicates outliers

Figure 4 shows the dispersion of the Dice-scores for active tumor per patient over 20 runs. Sixteen out of 21 patients have a median Dice-score of at least 60%. Fifteen patients have a lower quartile Dice-score of at least 50%. Seventeen patients have an interquartile range not higher than 15%. Seven patients had at least one run with a Dice-score lower than 30%. A large spread in the Dice-scores is seen for patient 6, who has a non-enhancing anaplastic astrocytoma with focal areas of enhancing GBM. Variability in the results was caused by the difficult differentiation of non-enhancing tumor from edema. Patient 8 suffers from a non-enhancing bifocal GBM, with only a small enhancing area in the smaller lesion. Due to the random selection of voxels in the active tumor region, in some runs voxels were only selected from one of both lesions, resulting in a failure to detect the other lesion. A large dispersion in the Dice-scores is also found for patient 18, exhibiting a heterogeneous and irregularly shaped GBM with varying degrees of enhancement in the active tumor region. Active tumor and necrosis were not well differentiated in some runs, mainly due to ambiguous voxel selection near the pathological tissue boundaries. The low outlier scores for patient 10 are due to the difficult differentiation of non-enhancing tumor from edema. The low outlier score for patient 13 is explained by a suboptimal and unrepresentative random voxel selection in the active tumor region.

**Fig. 5:**
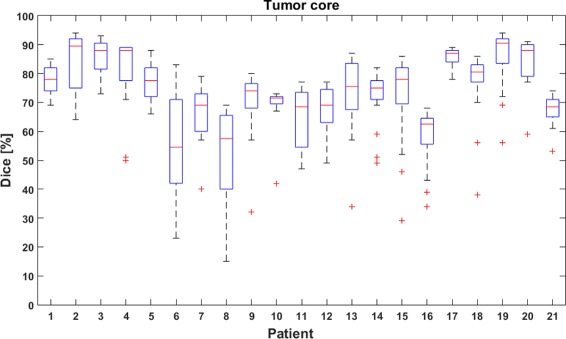
Boxplots showing the dispersion of the Dice-scores for the tumor core. Boxplots show quartile ranges of the Dice-scores, ’+’ indicates outliers

Boxplots for the tumor core are shown in Fig. 5. Eighteen out of 21 patients have a median Dice-score higher than 65%. For 17 patients, the lower quartile Dice-score was at least 60%. The interquartile range was not higher than 15% for 17 patients. Seven patients have at least one run with a Dice-score lower than 40%. For 5 out of the 7 patients, these low Dice-scores were found to be outliers (i.e. at a distance of more than 1.5 times the interquartile range from the lower quartile value).

**Fig. 6:**
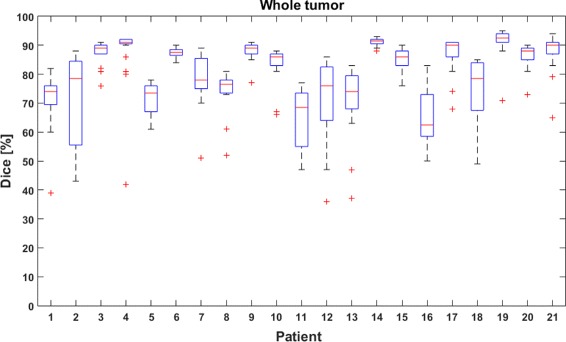
Boxplots showing the dispersion of the Dice-scores for the whole tumor region. Boxplots show quartile ranges of the Dice-scores, ‘+’ indicates outliers

Figure 6 shows the boxplots for the whole tumor region. A median Dice-score higher than 70% was found for 19 out of 21 patients. Eighteen patients have lower quartile value higher than 60%. Five patients have at least one run with a Dice-score lower than 45%. For 4 out of these 5 patients, such low values were found to be outliers.

Table 2 gives a comparison of the mean Dice-scores over all patients for the different NMF analyses. Overall, the best performance is obtained with $\mathbf {NMF_{\text {spatial\_sparse}}}$. When considering only spatial regularization (**N** **M** *F*_spatial_), segmentation results are mainly worse for the active tumor region, where the Dice-scores are even lower than for $\mathbf {NMF_{\text {no\_reg}}}$. The spatial smoothing was found to be too severe for several GBMs with a narrow ring-enhancing active tumor compartment. Compared to $\mathbf {NMF_{\text {no\_reg}}}$, Dice-scores are higher with $\mathbf {NMF_{\text {spatial\_sparse}}}$ for active tumor and the tumor core, with an increase of 2% in most cases. The Dice-score for the whole tumor region is 0 to 1% higher for $\mathbf {NMF_{\text {spatial\_sparse}}}$ compared to $\mathbf {NMF_{\text {no\_reg}}}$. $\mathbf {NMF_{\text {spatial\_sparse}}}$ does not show any improvement compared to **N** **M** *F*_cMRI_ for the active tumor region. An increase in Dice-score of 1 to 2% is found for the tumor core and an increase of 3 to 4% for the whole tumor region. The lowest Dice-scores are found for $\mathbf {NMF_{\text {no\_postproc}}}$, with a decrease of 6 to 8% compared to $\mathbf {NMF_{\text {spatial\_sparse}}}$.

**Table 2:** Mean Dice-scores [%] for NMF without regularization, with spatial regularization, with spatial and sparse regularization but without morphological post-processing, with spatial and sparse regularization on the cMRI data only, and with spatial and sparse regularization on the full MP-MRI dataset

	Active tumor [%]			Tumor core [%]			Whole tumor [%]		
	25_*th*_ prcntile	Mean	75_*th*_ prcntile	25_*th*_ prcntile	Mean	75_*th*_ prcntile	25_*th*_ prcntile	Mean	75_*th*_ prcntile
$\mathbf {NMF_{\text {no\_reg}}}$	58	63	72	66	72	79	76	80	84
**N** **M** *F*_spatial_	55	60	70	65	71	79	76	80	85
$\mathbf {NMF_{\text {no\_postproc}}}$	54	59	67	62	68	73	71	73	77
**N** **M** *F*_cMRI_	60	65	74	67	72	79	73	77	82
$\mathbf {NMF_{\text {spatial\_sparse}}}$	60	65	74	68	74	80	77	80	85

For each tissue class, the first column reports the mean of the 25^*th*^ percentile Dice-score across all patients, the second column the mean of the mean Dice-score across all patients and the third column the mean of the 75^*th*^ percentile Dice-score

Table 3 reports the mean Hausdorff distances over all the patients for the different NMF analyses. The lowest Hausdorff distances are found for $\mathbf {NMF_{\text {spatial\_sparse}}}$, with mean values of 6.1 mm, 7.4 mm and 8.2 mm for active tumor, the tumor core and the whole tumor region, respectively. Comparable but slightly higher Hausdorff distances are found for $\mathbf {NMF_{\text {no\_reg}}}$ and **N** **M** *F*_spatial_. Higher Hausdorff distances are found for **N** **M** *F*_cMRI_, with mean values of 7.4 mm, 9.1 mm and 14.1 mm. Hausdorff distances increase considerably when morphological post-processing is omitted: mean values of 26.6 mm, 29.3 mm and 28.9 mm are found for $\mathbf {NMF_{\text {no\_postproc}}}$.

**Table 3:** Mean Hausdorff distances [mm] for NMF without regularization, with spatial regularization, with spatial and sparse regularization but without morphological post-processing, with spatial and sparse regularization on the cMRI data only, and with spatial and sparse regularization on the full MP-MRI dataset

	Active tumor [mm]			Tumor core [mm]			Whole tumor [mm]		
	25_*th*_ prcntile	Mean	75_*th*_ prcntile	25_*th*_ prcntile	Mean	75_*th*_ prcntile	25_*th*_ prcntile	Mean	75_*th*_ prcntile
$\mathbf {NMF_{\text {no\_reg}}}$	3.3	6.9	8.1	3.1	7.9	9.7	3.8	8.6	10.6
**N** **M** *F*_spatial_	3.7	7.1	8.2	3.2	7.9	9.5	3.3	8.4	10.4
$\mathbf {NMF_{\text {no\_postproc}}}$	21.4	26.6	33.2	24.5	29.3	35.6	23.8	28.9	33.7
**N** **M** *F*_cMRI_	4.2	7.4	8.2	5.6	9.1	10.5	5.2	14.1	17.9
$\mathbf {NMF_{\text {spatial\_sparse}}}$	2.7	6.1	6.7	2.7	7.4	9.2	3.1	8.2	10.1

For each tissue class, the first column reports the mean of the 25^*th*^ percentile Hausdorff distance across all patients, the second column the mean of the mean Hausdorff distance across all patients and the third column the mean of the 75^*th*^ percentile Hausdorff distance

## Discussion

Comparison to other segmentation studies is sometimes hampered by the fact that different segmentation metrics or a different definition of the pathological subregions are being considered. We have quantified our results for active tumor, the tumor core and the whole tumor region using the Dice-score, in accordance with the Multimodal Brain Tumor Segmentation (BRATS) challenge held at the Medical Image Computing and Computer Assisted Intervention (MICCAI) conference. Looking at the Dice-scores reported for HGGs on the BRATS 2012 and 2013 datasets [14], it can be seen that our results are competitive, even when looking at the mean of 25^*th*^ percentile values (see Table 2). For the whole tumor region, Dice-scores are close to or even higher than 80%, which is in the range of inter-observer variability [14]. We have also applied semi-automated L1-regularized NMF directly to the BRATS 2013 Leaderboard dataset, allowing for a direct comparison with state-of-the-art [45]. It was found that our segmentation framework outperformed all other methods in segmenting the active tumor region, and was also competitive for the tumor core and the whole tumor region. As the BRATS dataset only contains cMRI data, comparison is most appropriate to the **N** **M** *F*_cMRI_ results. The inclusion of additional MRI modalities for brain tumor segmentation has been commonly suggested [2, 46] and initial studies have found improved segmentation results with extended MP-MRI datasets [28, 47]. When including ADC and rCBV into the MRI feature set, we found no advantage for the active tumor region, but Dice-scores increased by 1 to 2% for the tumor core and by 3 to 4% for the whole tumor region. These findings are in accordance with [28], where inclusion of PWI and DWI were found to be mainly advantageous for the tumor core and the whole tumor region. Segmentation performance was also assessed using the Hausdorff distance (see Table 3). As for the Dice-score, we obtained mean Hausdorff distances which are competitive with the best methods reported on the BRATS 2012 and 2013 datasets [14]. Another important consideration of any segmentation algorithm is its computational cost. To reduce computation time, we have applied downsampling to the in-plane dimensions of the imaging data. A downsampling factor of 2 was found not to affect segmentation performance. In the case of isotropic imaging data, where the out-of-plane resolution is as high as the in-plane resolution, downsampling could be applied to all 3 dimensions, with no expected loss in segmentation performance.

Most of the unsupervised brain tumor segmentation algorithms in literature incorporate prior knowledge in the form of basic assumptions or heuristics, which often limits their general applicability to a subset of gliomas or to a particular set of MP-MRI data. Prior knowledge in the form of user input is flexible, allowing to incorporate patient-specific information regarding appearance, location and/or shape of the tumor. Using our semi-automated framework, we were able to tackle the main limitations of many unsupervised classification methods like NMF. Most unsupervised algorithms require proper initialization to come to a valid locally optimal solution. Several studies cope with this by running the segmentation algorithm numerous times with a randomized initialization, then selecting the final solution based on a predefined objective function [24, 28]. We are combining user-defined voxel selection for initializing the pathological sources with a sophisticated initialization of the normal brain tissue sources based on SPA and FCM. As SPA is sensitive to outliers, its output is fed into the FCM algorithm, thereby providing a deterministic initialization for FCM.

Due to the lack of labelled training data, automatic assignment of a tissue label to each segmented region is non-trivial for unsupervised methods. Several studies do not explicitly propose an automated labelling strategy [25, 27], while others assume specific characteristics of the tumor compartments in specific MRI images (such as e.g. contrast enhancement of active tumor) [22, 23]. In our proposed methodology, tissue labelling automatically results from the voxel selection in the pathological regions.

False positive regions were excluded from the pathological tissue masks by exploiting the spatial location of the selected voxels in a morphological post-processing procedure. As the pathological tissue classes are assumed to form spatially consistent regions, only the connected component closest to each selected voxel is withheld in the segmentation masks. Loss of tumor components not selected by the user is avoided by assuming spatial adjacency of the pathological regions. Mean Dice-scores decreased by 6 to 8% when omitting the morphological post-processing step after NMF with spatial regularization and sparseness (Table 2, $\mathbf {NMF_{\text {no\_postproc}}}$). Loss in performance was more pronounced when considering the Hausdorff distance, with mean values being an order of magnitude larger for $\mathbf {NMF_{\text {no\_postproc}}}$ than for the other NMF analyses (Table 3). This indicates that our semi-supervised approach allows for the removal of false positive regions at considerable distance from the actual tumor volume, by exploiting the spatial location of the user-defined seeding points. Similar types of post-processing have been proposed to enhance segmentation results. Cordier et al. only withheld 1 or 2 connected components for each tissue region based on their size, assuming that the largest component(s) correspond to the true tissue region [32]. Menze et al. exploit knowledge about shape and location of the tumor based on an atlas tissue prior to remove false positives [25]. Havaei et al. initialize a k-nearest neighbors (kNN) classifier based on voxel selection by the user [35]. They do not exploit the spatial location of the selected voxels in a post-processing step, but instead they add voxel coordinates to the feature set of the kNN classifier.

Nowadays, a common approach to further improve segmentation results is to model the spatial dependency between the tissue labels of adjacent voxels, typically using a Markov Random Field (MRF) [25, 35] or a Conditional Random Field (CRF) [15, 17]. With MRF, a fixed penalty term is added to the cost function when adjacent voxels have different labels. This penalty term is also depending on the (dis)similarity of the feature vectors when using CRF. To the authors’ knowledge, this is the first study to consider spatially regularized NMF for tumor segmentation. We have added a spatial regularization term with sparseness to the NMF objective function. L1-regularization was used to promote piece-wise smoothness of the abundance maps, allowing for discontinuities at the tissue boundaries. Whereas MRF and CRF are directly applied to the voxel labels, we applied L1-regularization to the tissue abundance maps which represent continuous variables. In some patients, suboptimal segmentation results were found when using spatial regularization only. This is reflected in the lower Dice-scores for **N** **M** *F*_spatial_ in the active tumor region and in the tumor core region (Table 2). The original abundance maps have a relatively low degree of sparseness, such that piece-wise spatial smoothness was not always restricted to the true tissue boundaries. Results were improved by combining spatial regularization with sparseness. This kind of regularization resembles MRF more closely, since MRF implies absolute sparseness of the tissue labels. Over all patients, we found an increase in mean Dice-score by 2% for active tumor and for the tumor core when using spatial regularization with sparseness (Table 2). For the whole tumor region, an increase of 1% was found for the 25^*th*^ and 75^*th*^ percentile Dice-scores and no increase was found for the mean value. Similar improvements in segmentation results have been reported in the literature [17, 35].

As imaging characteristics vary considerably across glioma subtypes and grades, supervised classification methods require extensive training datasets with a uniform acquisition protocol. To increase specificity, separate classifiers are usually built for low- and high-grade gliomas [14]. Using training data from different patients to segment a new test case requires careful calibration of image intensities across different patients, which is non-trivial in the presence of pathology. Our method uses labelled data only from the patient under study. Feature vectors averaged over the local neighborhood of the selected voxels are assumed to be approximative prototypes of the pathological tissue regions to be segmented.

**Fig. 7:**
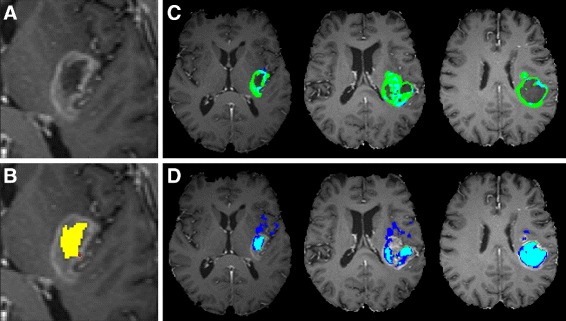
Example of a bad segmentation result due to suboptimal voxel selection. A close-up of a GBM lesion on an axial T1C slice (**a**). The manually segmented necrotic region is shown in *yellow* (**b**), the selected necrotic voxel is marked in red. Segmentation of the active tumor region (**c**) and necrotic region (**d**) on several slices, *blue* indicates NMF segmentation, *green* indicates manual segmentation and cyan indicates overlap

One of the main limitations of any semi-automated segmentation algorithm is that they don’t provide reproducible results. Robustness against user input variability is therefore a key aspect. We have assessed user input variability by repeatedly selecting random voxels in each pathological region. Fairly robust segmentation results were found for most patients (see Figs. 4, 5 and 6), but a large spread in the Dice-scores and low outlier values were found in several patients as well. In some cases suboptimal results could be explained by ambiguous voxel selection near the tissue boundaries. Figure 7 illustrates a case of suboptimal voxel selection for patient 1. One of the necrotic voxels was selected at the edge of the manually segmented necrotic region (red voxel in B, other necrotic voxels were selected on other slices). This voxel is located very near the active tumor region and does not show the hypo-intense T1C signal which is characteristic for necrosis. The resulting NMF segmentation is shown in C for active tumor and in D for necrosis. The necrotic voxel selected at the boundary results in an overestimation of the necrotic region and an underestimation of the tumor region. The low outlier Dice-score for patient 1 for active tumor in Fig. 4 corresponds to this voxel selection. Another patient had a bi-focal tumor, and in some runs only one of both lesions was detected as no voxels were selected in the other lesion. Random voxel selection from the manually segmented tumor subregions might not be entirely representative for the user input variability that is to be be expected from trained radiologists. It might be more effective to consider actual user input from various experts in a future study. Valid segmentation results can only be obtained when seeding points are selected in an intelligible way. Spatially distributing the selected voxels is one way of covering intra-tissue heterogeneity. Seeding points should be selected in each lesion in order to properly detect multi-focal tumors. Careful voxel selection is expected to further improve segmentation results and reduce variability.

## Conclusion

We have presented a semi-automated brain tumor segmentation method, based on NMF with L1-regularization to promote spatial consistency and sparseness of the tissue abundance maps. The semi-automated framework was applied to an MP-MRI dataset consisting of cMRI, PWI and DWI. User-defined voxel selection is applied to initialize pathological sources of the NMF analysis, and to exploit knowledge about the spatial location of the tumor to remove false positives in a post-processing step. In this way, we aimed at incorporating prior knowledge while maintaining general applicability of our method to any type of glioma and to any MP-MRI dataset. Sensitivity to user input variability was explored through repeated analyses with different voxel selection. Robust results were found for most patients, although careful voxel selection is mandatory to avoid sub-optimal segmentation. Based on the reported mean Dice-scores and Hausdorff distances, segmentation results are competitive with state-of-the-art in literature.

## Abbreviations

ADC: Apparent diffusion coefficient
BRATS: Multimodal Brain Tumor Segmentation challenge
cMRI: Conventional MRI
CRF: Conditional random field
DWI: Diffusion-weighted imaging
FCM: Fuzzy C-means clustering
FLAIR: Fluid-attenuated inversion recovery
GBM: Glioblastoma multiforme
GNDL: Gauss-Newton with dogleg trust region
HGG: High-grade glioma
kNN: k-nearest neighbors
MP-MRI: Multi-parametric MRI
MRF: Markov random field
NMF: Non-negative matrix factorization
PWI: Perfusion-weighted imaging
RANO: Response assessment in neuro-oncology
rCBV: Relative cerebral blood volume
SPA: Successive projection algorithm
T1C: T1-imaging with contrast enhancement
TE: Echo time
TI: Inversion time
TR: Repetition time
